# *Blastobotrys adeninivorans* and *B. raffinosifermentans*, two sibling yeast species which accumulate lipids at elevated temperatures and from diverse sugars

**DOI:** 10.1186/s13068-019-1492-x

**Published:** 2019-06-20

**Authors:** Stéphane Thomas, Daniel R. A. Sanya, Florian Fouchard, Huu-Vang Nguyen, Gotthard Kunze, Cécile Neuvéglise, Anne-Marie Crutz-Le Coq

**Affiliations:** 1 Micalis Institute, INRA, AgroParisTech, Université Paris-Saclay, 78350 Jouy-en-Josas, France; 2 Leibniz Institute of Plant Genetics and Crop Plant Research (IPK), Correnstr. 3, 06466 Gatersleben, Germany

**Keywords:** Microbial oil, Lipid metabolism, Biotechnology, Saccharomycotina, Thermotolerance, Oleaginous yeasts

## Abstract

**Background:**

In the context of sustainable development, yeast are one class of microorganisms foreseen for the production of oil from diverse renewable feedstocks, in particular those that do not compete with the food supply. However, their use in bulk production, such as for the production of biodiesel, is still not cost effective, partly due to the possible poor use of desired substrates or poor robustness in the practical bioconversion process. We investigated the natural capacity of *Blastobotrys adeninivorans*, a yeast already used in biotechnology, to store lipids under different conditions.

**Results:**

The genotyping of seven strains showed the species to actually be composed of two different groups, one that (including the well-known strain LS3) could be reassigned to *Blastobotrys raffinosifermentans*. We showed that, under nitrogen limitation, strains of both species can synthesize lipids to over 20% of their dry-cell weight during shake-flask cultivation in glucose or xylose medium for 96 h. In addition, organic acids were excreted into the medium. LS3, our best lipid-producing strain, could also accumulate lipids from exogenous oleic acid, up to 38.1 ± 1.6% of its dry-cell weight, and synthesize lipids from various sugar substrates, up to 36.6 ± 0.5% when growing in cellobiose. Both species, represented by LS3 and CBS 8244^T^, could grow with little filamentation in the lipogenic medium from 28 to 45 °C and reached lipid titers ranging from 1.76 ± 0.28 to 3.08 ± 0.49 g/L in flasks. Under these conditions, the maximum bioconversion yield (*Y*_FA/S_ = 0.093 ± 0.017) was obtained with LS3 at 37 °C. The presence of genes for predicted subunits of an ATP citrate lyase in the genome of LS3 reinforces its oleaginous character.

**Conclusions:**

*Blastobotrys adeninivorans* and *B. raffinosifermentans,* which are known to be xerotolerant and genetically-tractable, are promising biotechnological yeasts of the Saccharomycotina that could be further developed through genetic engineering for the production of microbial oil. To our knowledge, this is the first report of efficient lipid storage in yeast when cultivated at a temperature above 40 °C. This paves the way to help reducing costs through consolidated bioprocessing.

**Electronic supplementary material:**

The online version of this article (10.1186/s13068-019-1492-x) contains supplementary material, which is available to authorized users.

## Background

The use of renewable feedstocks to produce microbial oil for biodiesel or other applications has become a scientific and environmental issue over the last two decades. Oleaginous yeasts, defined as those that can accumulate lipids to over 20% of their dry-cell weight (DCW), are still being screened and can be found in different taxa. A useful criterion is their ability to grow on pentoses or polysaccharides, which are lignocellulosic sugars. However, although some of these yeasts are immediately suitable for providing useful enzymes (such as sugar hydrolases), their capacity to be custom engineered for specific lipid production will require additional effort. Conversely, known genetically-tractable yeasts may not have yet been tested for their oleaginous potential.

*Blastobotrys adeninivorans* is part of a basal clade of the Saccharomycotina subphylum [1] and diverged long before *Saccharomyces cerevisiae*. The species was first described in 1984, under the name of *Trichosporon adeninovorans* [2], and successively reclassified into the genera *Arxula* (*adeninivorans*) in 1990 [3] and *Blastobotrys* in 2007 [1]. A few natural isolates of soil, plant (wood hydrolysate, silage), or animal origin have been described in the literature or deposited in culture collections. Among them, the type strain CBS 8244^T^ and the industrial strain LS3 have been the best studied, LS3 being used to develop auxotrophic recipients for genetic transformation [4, 5].

Since the early 1990s, the species has attracted attention because of its interesting biochemical, physiological, and genetic properties. First, it exhibits a versatile metabolism, indicative of the presence of various degradative enzymes and pathways linked to central metabolism [6]. One to several strains can assimilate a number of sugar substrates, such as d-galactose, d-xylose, l-arabinose, raffinose, sucrose, trehalose, cellobiose, starch, and arbutin, and can also ferment some of them (glucose, sucrose, and starch) [6, 7]. Organic acids (e.g., pyruvate, acetate, and butyrate), as well as aromatic compounds (e.g., hydroxybenzoates), may also serve as a growth source [6, 7]. Moreover, various nitrogenous compounds may serve as nitrogen sources (e.g., urea, formamide, ethanolamine, and most amino acids), both carbon and nitrogen sources (e.g., adenine, acetamide, uric acid, putrescine, and some amino acids) [6, 7], or an energy source via nitrate reductase [8]. Second, *B. adeninivorans* exhibits extremophilic traits, such as halotolerance, osmotolerance, and thermotolerance. This last characteristic is particularly interesting, as only a few yeasts, especially few biotechnological yeasts, have been found to grow at temperatures over 40 °C [9, 10]. Indeed, *B. adeninivorans* is able to grow at up to 48 °C [11] and survive a few hours at 55 °C [12]. In addition, as shown in the case of the LS3 strain, temperature can induce a reversible developmental state, with the cells being mainly in the yeast form at lower temperatures and (pseudo)hyphae forms at higher temperatures (above 42 °C) [11]. Third, with an eye towards metabolic engineering, valuable information has been brought to light by sequencing of the complete genome of LS3 [13] and integrative plasmids and recipient strains for genetic engineering have been developed [14]. The small size of the haploid genome of this yeast (11.8 Mb) is an additional advantage for gene function studies or chassis strain development for various applications.

*Blastobotrys adeninivorans* has earned its stripes as a biotechnological yeast mainly after being engineered in the laboratory of Kunze. It has been engineered for diverse applications, such as for a biosensor, a platform for protein expression, and a solventogenic yeast for butanol production [14]. Efficient cultivation in bioreactors has also been achieved, with Stöckmann et al. reporting a biomass titer of 240 g/L under pressure [15]. The ability of *B. adeninivorans* to produce lipids has been rarely reported in the literature. Strain LS3 was included in an assay to monitor lipid bodies by impedance spectroscopy a decade ago [16]. More recently, Olstorpe et al. [17] reported the relative fatty acid (FA) composition of two strains of this species, without however mentioning the total amount of lipid.

Several yeast species are well-known lipid producers. Three have attracted most of the research efforts in recent years [18]: *Yarrowia lipolytica* and *Lipomyces starkeyi*, belonging to the Saccharomycotina, and *Rhodotorula* (*Rhodosporidium*) *toruloides*, belonging to the Basidiomycota. All three species exhibit a genome size of over 20 Mb [19–21]. *L. starkeyi and R. toruloides* are known for their utilization of a wide range of substrates and their outstanding lipogenic capabilities (up to 70% DCW as lipids) [22–24]. *L. starkeyi* is particularly well suited for rapid and simultaneous utilization of sugar mixtures, reaching a lipid content of 52% DCW and a lipid titer of 13.3 g/L in a mixture of cellobiose and glucose [25]. Strain diversity or culture conditions may alter lipid production; to maximize lipid production, the synthesis of endopolysaccharides under nitrogen limitation should be avoided [26]. Although progress has been made in recent years, the genetic engineering of these yeasts is still a shortcoming [18]. *B. adeninivorans* is phylogenetically close to *Y. lipolytica* [27]. Some strains of *Y. lipolytica*, as well as other species in the genus, are naturally more prone to store lipids from exogenous hydrophobic compounds than to turn glucose into lipids. Lipid content can easily reach 30% DCW [28] in the former case and generally 6 to 15% DCW in the latter [28, 29]. Metabolic engineering and/or process optimization, as well as strain screening, have been successfully used to increase lipid content above 35% DCW and reportedly up to 90%. *Y. lipolytica* has also required genetic engineering or specific culture conditions to utilize xylose, cellobiose, or cellulose polymers, which are major components of lignocellulosic materials. Depending on the strain, biotechnological processes should be fine-tuned to prevent excessive excretion of citric acid, which competes with lipid production in the presence of excess carbon, and strong filamentation, which depends on multiple parameters (dissolved oxygen, medium composition, and initial carbon concentration, etc.) and may alter the process of lipid production and its yield [30, 31].

None of these species has been reported to grow at temperatures above 37 °C, though some thermotolerant strains may have been isolated. An *R. toruloides* DMKU3-TK16 mutant obtained through an adaptive breeding strategy can store lipids at approximately 14% DCW at 37 °C [32]. However, working at upper temperatures could provide specific advantages for compound solubilization (e.g., FA), help to minimize the costs of cooling of bioreactors and facilitate consolidated bioprocesses. These processes are considered to be relevant at a temperature around 50 °C as microbial enzymes involved in the degradation of hemicellulose/cellulose or starch have been generally described to have optimal temperatures above 40 °C, up to 60 °C [10, 33, 34].

The upper permissive temperature for growth of eucaryotes has been considered to be near 60 °C [35], far below that of eubacteria and archae. Relatively few thermophilic fungi were found to grow at elevated temperatures, optimally around 55 °C, and tolerated up to 61 °C [36, 37]. Some of these (not yeast-like) fungi might be useful bioconverters of lignocellulosic residues into sugars [37]. Yeast species comprising strains able to grow at temperatures up to 45 °C were listed in a specific survey by Robert et al. in 2015 [9]. Among the 28 most thermotolerant species, two are well-known for different applications not related to lipid production, *Ogataea* (*Hansenula*) *polymorpha* and *Kluyveromyces marxianus* [38]. Few others were reported with a lipid content over 20% DCW in screening studies, such as *Kodamaea ohmeri* [39] and *Kurtzmaniella cleridarum* [40]. We are not aware of studies concerning lipid accumulation at high temperatures, except for *K. marxianus*. The potential of *K. marxianus* as an oleaginous yeast is unclear. A strain isolated from Kefir exhibited over 30% DCW in lipids [41], while different strategies—including genetic modifications typically used in other organisms—to increase lipogenesis of several *K. marxianus* strains yielded at best a FA content of 16% DCW [42].

Because of its aforementioned advantageous traits, we reasoned that *B. adeninivorans* could be a valuable thermotolerant oleaginous yeast. Here, we assessed the ability of *Blastobotrys* species to naturally produce and accumulate lipids from a set of different substrates. Several strains of what was believed to be *B. adeninivorans* were used to examine the robustness of lipid production in the species. However, in the course of our study, strain genotyping showed that they can be divided into two different groups and various marker sequences actually placed the LS3 group into the *Blastobotrys raffinosifermentans* species. We took advantage of the thermotolerance of these yeasts and assayed lipid production at various temperatures, up to 45 °C. Overall, the natural capacity of these two species to produce lipids is promising and may be an alternative to currently-studied oleaginous yeasts.

## Results and discussion

### Nitrogen limitation and lipid accumulation in strain LS3

Oleaginous yeasts generally synthesize and abundantly store lipids in lipid droplets under specific environmental conditions, one of which is nitrogen limitation [43, 44]. In addition to FA synthesis, these yeasts can import and direct exogenous FAs into the lipid storage pathway, sometimes with particularly high efficiency, such as in *Y. lipolytica* [45].

**Fig. 1:**
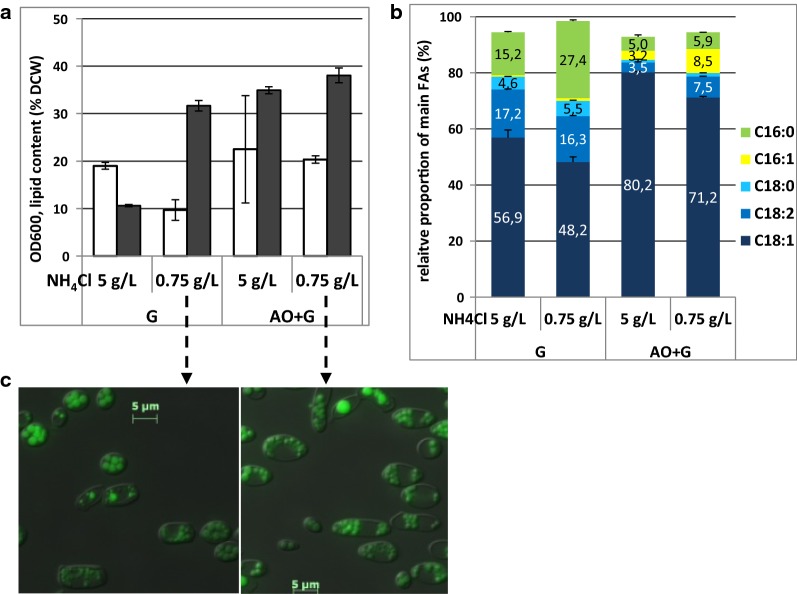
Effect of limiting nitrogen on the synthesis and accumulation of lipids in strain LS3. LS3 was cultivated for 72 h in YNB-based medium with carbon source at 30 g/L. The concentrations of 5 g/L and 0.75 g/L NH_4_Cl led to a C/N ratio of 90 and 60, respectively, for cultures in glucose (G) and to a C/N ratio of 16 and 106, respectively, for cultures in a mixture of oleic acid and glucose (AO + G). **a** Cell density is expressed as OD_600_ (white bars) and total lipid content as the percentage of dry-cell weight (black bars). Average values and standard deviations (*n* = 2) are shown in the histograms. **b** Relative proportion (%) of main FAs in each FA profile. **c** BODIPY-stained lipid droplets in cells sampled from low-nitrogen cultures in glucose (left) or in the presence of oleic acid (right)

We examined the capacity of strain LS3 to store lipids after neo-synthesis from glucose or after accumulation from exogenous oleic acid. We performed flask cultivation in YNB-based media with two different carbon sources at a fixed concentration of 30 g/L (glucose or a mixture of oleic acid and glucose) and two different concentrations of NH_4_Cl as a unique nitrogen source (5 g/L or 0.75 g/L) to vary the carbon-to-nitrogen ratio (C/N). Efficient lipid storage occurred following growth in both substrates to above 30% DCW. However, as expected, it strongly relied on a low-nitrogen concentration when glucose was the sole substrate (Fig. 1a). When the exogenous FA C18:1 was provided, the FA profile of the strain was enriched in C18:1 (Fig. 1b), suggesting entry of the FA directly into the storage pathway. BODIPY staining showed storage to occur in the form of neutral lipids in lipid droplets in both cases (Fig. 1c). Our results show that strain LS3 has a good natural capacity for both lipogenesis and lipid accumulation upon FA uptake; in reaching approximately 30% DCW as lipids from glucose, it may be qualified as oleaginous.

### The genome of strain LS3 encodes a putative ATP citrate lyase

The complete genome sequence of LS3 (first identified as *B. adeninivorans*, then reassigned to *B. raffinosifermentans*; see below and Table 1), already recognized to be of biotechnological interest, was published a few years ago [13]. We searched the genome for potential “markers” of oleaginicity by mining the genome of LS3 for specific genes involved in lipid metabolism, using proteins of *Y. lipolytica* as a query (Table 2). First, cytoplasmic ATP citrate lyase (ACL), which can efficiently convert respiratory citrate to acetyl-CoA for FA biosynthesis, is considered to be a signature (first biochemical then genetic) of oleaginous yeasts [46–48]. ACL, of which the subunits are encoded by two distinct genes in LS3, could be confidently predicted by its high amino acid similarity with this enzyme in *Y. lipolytica* (Table 2). The presence of these genes suggests the native oleaginous character of the *Blastobotrys* strain. Second, we found that LS3 possesses a copy of an *AAL*-like gene encoding putative peroxisomal acyl-CoA synthetase (Table 2). Homologous gene products in *Y. lipolytica*, which has a family of ten genes of Acyl/Aryl-CoA-ligases (AAL), were shown to be responsible for the activation of FAs into acyl-CoA [48]. Although various types of acyl-CoA synthetases are found in yeast (e.g., Faa2 in *S. cerevisiae*), the distribution of these particular *AAL*-like genes was hypothesized to be biased towards oleaginous species [48]. Third, we examined the formation of triacylglycerol (TG). In yeast, TG synthesis relies on esterification of diacylglycerol with a third FA [49]. This last step of TG formation has been considered to be a limiting step for oil storage. It involves acyl-CoA dependent diacylglycerol acetyltransferases (DGAT) and/or phospholipid:diacylglycerol acetyltransferases (PDAT), which use phospholipids as FA donors. *S. cerevisiae*, *Y. lipolytica* [50], and *B. raffinosifermentans* LS3 (Table 2) all possess one gene encoding a PDAT enzyme. *S. cerevisiae* possesses only one DGAT gene, whereas *Y. lipolytica* [51, 52] and LS3 (Table 2) have two DGATs, possibly emphasizing their capacity for TG storage, a preferred form of oil storage in oleaginous yeasts. While our study was being conducted, the genetic content of strain CBS 8244^T^ (type strain of *B. adeninivorans*) was analyzed in a patent in view of lipid production [53]. The genome reportedly contained the two genes for ACL and two different genes for DGAT, as in LS3. The genome sequence is not available, however.

**Table 1:** List of strains, received as *Blastobotrys adeninivorans* and reassigned to two different species

Strain	Synonym	Isolated from	By	Reference	New species assignment
CBS 8244^T^	CSIR 577, CLIB 1468	Soil, The Netherlands (adenine-enrichment)	Middelhoven, 1983	[2]	*B. adeninivorans* (type strain)
CBS 7766		Reptile (liver and intestines), Sweden	Mattsson, 1993		*B. adeninivorans*
CBS 7350		Maize silage, The Netherlands	Middelhoven, 1987		*B. adeninivorans*
LS3		Wood hydrolysates, Russia (Siberia)	Kapultsevich, selected as industrial strain	[9]	*B. raffinosifermentans*
CBS 8335		Soil (clay-like, pH 8.5), Italy	Middelhoven 1996		*B. raffinosifermentans*
CBS 7370	CSIR 1117	Soil (humus-rich), South Africa	van der Walt	[3]	*B. raffinosifermentans*
CBS 7377	CSIR 1118	Soil, South Africa	van der Walt, 1988	[3]	*B. raffinosifermentans*

*CSIR* Council for Scientific and Industrial Research (Pretoria, South Africa), *CLIB* CIRM-Levures (France)

**Table 2:** Selected genes of lipid metabolism in the genome of *B. raffinosifermentans* LS3

Gene identifier	Previous function/similarity [reference yeast]^a^	New function prediction	Amino acid % identity (coverage)	Process
ARAD1B07414	Succinyl CoA ligase (alpha subunit) [sc]	ATP:citrate lyase (subunit) EC:2.3.3.8	88% (99%) with YALI0E34793	Formation of cytosolic acetyl-CoA, precursor of FA synthesis
ARAD1D32164	Succinyl CoA ligase (beta subunit) [sc]	ATP:citrate lyase (subunit) EC:2.3.3.8	82% (100%) with YALI0D24431	Formation of cytosolic acetyl-CoA, precursor of FA synthesis
ARAD1C08250	Acyl-CoA:diacylglycerol acyltransferase (DGAT) [sc]	DGAT (no change) EC:2.3.1.20	69% (59%) with YALI0E32769	Formation of TG
ARAD1D42460	Acyl-CoA:sterol acyltransferase [sc]	DGAT EC:2.3.1.20	42% (86%) with YALI0D07986	Formation of TG
ARAD1C30118	Weakly similar to FAT2 peroxisomal AMP binding protein [sc]	Acyl-CoA synthetase	38% (99%) with YALI0E12419 (10 paralogs). PTS1 motif^b^	Formation of acyl-CoA (peroxisomal activation of FA)

^a^In the current (first) version of the annotated genome of LS3 in GRYC database. First-round annotation was performed using BLAST against *S. cerevisiae* (sc) as the reference yeast to look for similarity

^b^AKL in C terminus of the protein which likely targets it to peroxisome

### Diversity among strains and species assignment

We continued our investigation of the oleaginous character of the species by expanding our investigation to several strains. Strains LS3 and CBS 8244^T^ were previously suggested to belong to different genetic subgroups, given the size of their four chromosomes and DNA-fingerprints [54]. We thus genotyped five strains of various origins obtained from the CBS collection (Table 1) along with LS3 and CBS 8244^T^.

**Fig. 2:**
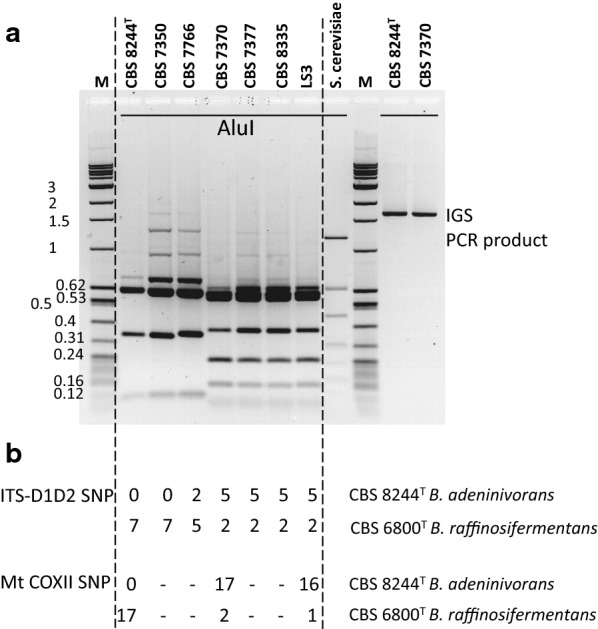
Two genotypic groups in strains previously identified as *B. adeninivorans* and relation with B. *raffinosifermentans.* **a** IGS profiles. The right part of the agarose gel shows an example of the PCR product obtained for two strains. The left part shows *Alu*I restriction profiles for the seven strains. *Saccharomyces cerevisiae* ATCC 42367 was used as a control. The M wells are occupied by the molecular weight standard, a mixture of NEB quick-load 1 kb ladder and pBR322 *Msp*I-digest, for which the size of some bands is indicated in kb. **b** Single-nucleotide polymorphisms (SNPs) in the ITS-D1D2 (1086 nt) and mitochondrial COXII (598 nt) sequences. The number of SNPs (including one indel in ITS) is indicated for these markers for each comparison between the strain above and the type strains to the right. (–) indicates not determined

Using *Alu*I fingerprinting of intergenic spacer rDNA (IGS), we found the seven strains to be clearly distributed into two distinct groups, a first group of three strains, including CBS 8244^T^, and a second group of four strains, including LS3 (Fig. 2a). The sequences of ITS and D1D2 showed the second group to be homogenous for this marker (no sequence polymorphism between strains), whereas it differs from the first group by three to five nucleotides (Fig. 2b, Additional file 1: Figure S1). The sequence of mtCOXII in CBS 7370 and LS3 confirmed the proximity of these strains, while showing divergence of CBS 8244^T^ (Fig. 2b). The sequence of these markers for strains of the LS3 group better matched those of *B. raffinosifermentans*, according to the YeastIP database [55]. This species, closely related to *B. adeninivorans*, was described in 2007 and represented by a unique strain at that time [1]. Our results highlight the existence of two distinct groups among strains previously identified as *B. adeninivorans*, resulting in the reassignment of the LS3 group to the *B. raffinosifermentans* species.

### Capacity of sugar assimilation of *B. raffinosifermentans* and *B. adeninivorans*

We examined the ability of the various strains to grow on three different simple carbohydrates, potentially useful as carbon and energy sources for microbial oil production. All strains exhibited relatively similar growth curves when cultivated in minimal medium supplemented with glucose or xylose, which reflects efficient assimilation of these substrates (maximum growth rate from 0.13 to 0.16 h^−1^ and stationary phase reached within 24 to 30 h in microplates). In contrast, glycerol promoted much slower and delayed growth, depending on the strain and substrate concentration (Additional file 1: Figure S2). For *B. raffinosifermentans* strains, the maximum growth rate ranged from 0.078 to 0.12 h^−1^ in 10 g/L glycerol and 0.025 (CBS 8335) to 0.1 h^−1^ in 2 g/L glycerol. This phase was generally preceded by a much slower growth phase or lag phase, especially in low concentrations of glycerol, extending up to 60 h. For strains of the *B. adeninivorans* species (CBS 8244^T^ group), growth was very weak (10 g/L glycerol) or not visible (2 g/L glycerol) after 96 h cultivation in microplates (Additional file 1: Figure S2). In plate assays with 10 g/L glycerol, growth was also reproducibly observed to be delayed relative to growth on glucose, and all seven strains reached lower-density streaks on glycerol than glucose after 7 days at 28 °C. As a control, *Y. lipolytica* W29 grew similarly on both glycerol and glucose plates (data not shown).

Glycerol assimilation is a typical variable trait among yeasts [56]. Glycerol is generally regarded to be an efficient carbon source for *L. starkeyi* [22] and even a preferred substrate for *Y. lipolytica*, especially as it is first consumed when both glycerol and glucose are present [26, 57]. In contrast, most strains of *S. cerevisiae* do not grow in synthetic glycerol media without successful adaptation or medium supplementation with complex compounds, amino acids, or nucleobases [56]. Our results underline a difference in glycerol catabolism between some oleaginous yeasts and the two *Blastobotrys* species under study, or at least in its regulation. More favorable environmental conditions or conditions of possible adaptation are yet to be determined for efficient glycerol assimilation. However, glycerol is not only a potential substrate for growth but also the precursor backbone esterified by FAs in triglycerides. Sugar/glycerol blends or glycerol co-feeding have been successfully used to improve lipogenesis [58, 59] and would be interesting to evaluate in this particular context of poor glycerol catabolism, providing that transport would not be a limiting step.

### Capacity for lipogenesis of *B. raffinosifermentans* and *B. adeninivorans*

**Fig. 3:**
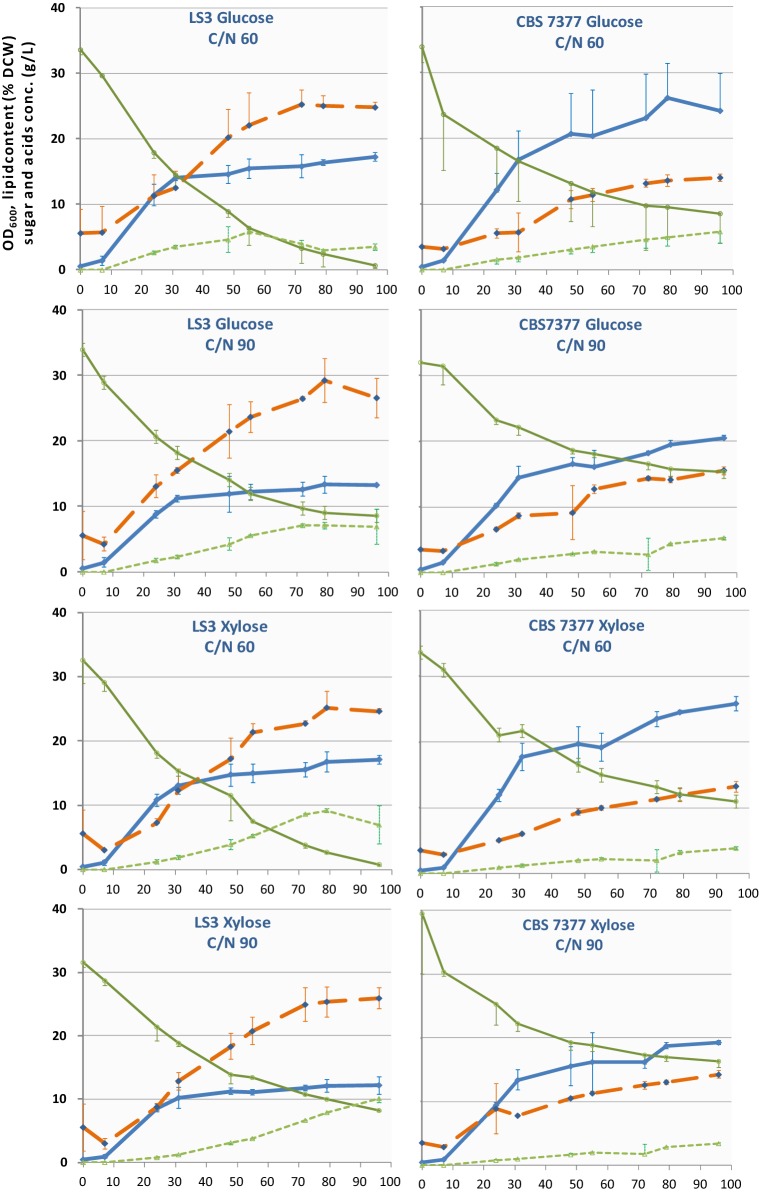
Bioconversion of sugars into lipids during the growth of two strains of *B. raffinosifermentans* in nitrogen-limited YNB-based media. Glucose and xylose were each used as the C source (30 g/L) at two different C/N ratios. Average values and the standard deviation (*n* = 3) for OD_600_ (blue line), residual sugar in the medium (g/L, plain green line), cumulative organic acids (g/L, dotted green line), and lipid content (% DCW, orange line) were plotted over time (h)

**Fig. 4:**
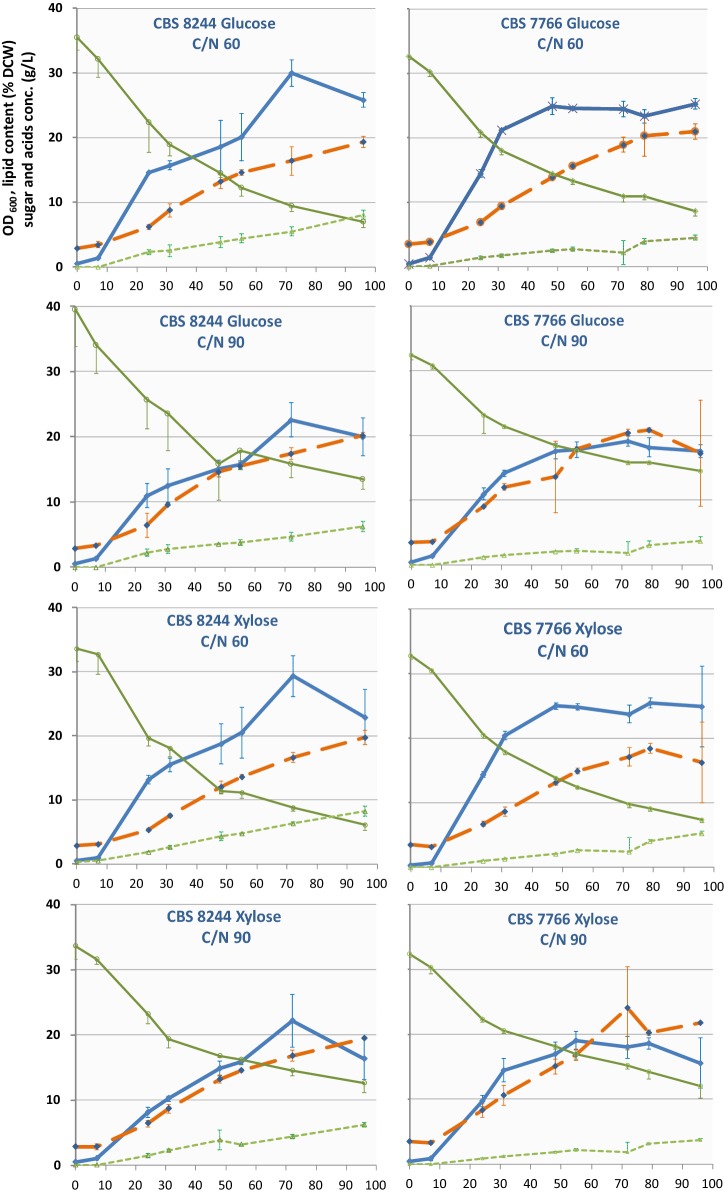
Bioconversion of sugars to lipids during growth of two strains of *B. adeninivorans* in nitrogen-limited YNB-based media. Glucose and xylose were each used as the C source (30 g/L)at two different C/N ratios. Average values and the standard deviation (*n* = 3) for OD_600_ (blue line), residual sugar in the medium (g/L, plain green line), cumulative organic acids (g/L, dotted green line), and lipid content (% DCW, orange line) were plotted over time (h)

We evaluated the robustness of lipogenesis among strains and investigated possible phenotypic diversity concerning this trait by assaying lipid production over time of two strains of each species (LS3 and CBS 7377 for one and CBS 8244^T^ and CBS 7766 for the other). They were cultivated in glucose or xylose, in which all strains grew easily. We periodically measured the OD_600_ and FA content of the cells, as well as sugars and organic acids in the medium, throughout the 96-h cultivation in flasks under nitrogen limitation (0.75 g/L or 0.5 g/L NH_4_Cl in YNB medium with 30 g/L carbon source, leading to a mass carbon-to-nitrogen ratio (C/N) of 60 and 90, respectively). Under these conditions, lipogenesis began at the start of growth for all strains. The highest amount of lipids in the cells was reached after 72 h and ranged from 14 to 29% DCW (Figs. 3, 4). Lipogenesis was negligibly better at C/N 90 than C/N 60 (up to 10% better) but it lowered growth and global sugar consumption.

The four strains exhibited different behaviors in terms of growth and metabolism, lipid accumulation, organic acid excretion, and sugar consumption. There were no strict species-specific correlations. CBS 7377 was the slowest and least efficient strain for lipid accumulation, whereas LS3 (of the same species) reached the highest lipid content. Conversely, the OD reached by LS3 in stationary phase was the lowest of all strains. However, different OD may reflect morphological differences between strains rather than differences in biomass production. The results of the strains of *B. adeninivorans* were more similar to each other. Overall, both species cultivated in low-nitrogen medium reached a lipid content above 20%. Sugar uptake was more efficient for LS3 than for other strains in all media (Figs. 3, 4). With few exceptions, < 75% of the initial sugar was consumed by the end of experiment, leaving room for optimization of the medium and culture conditions. Within the time of the experiment, *B. raffinosifermentans* LS3 reached the highest lipid content among all four strains, whereas *B. adeninivorans* CBS 8244^T^ accumulated lipid more slowly and probably did not reach its maximum.

The excretion of organic compounds is a well-known competing sink for lipid production, particularly that of citric acid, both an intermediate of the TCA cycle and a precursor for acetyl-CoA in oleaginous yeasts. In our shake-flask experiments, we found two different organic acids to be excreted into the medium: citric acid and acetic acid. Globally, their cumulative concentration in the medium (Figs. 3, 4) tended to steadily rise over time and reached 3.4 ± 0.2 g/L (CBS 7766 cultivated in xylose C/N 90) to 10.1 ± 0.6 g/L (LS3 cultivated in xylose C/N 90) by the end of experiment. LS3 was unique concerning the excretion of organic acids, both in terms of their total concentration, which did not increase after 50 to 60 h of cultivation in some media, and the relative proportion of acetic to citric acid. Although acetic acid was the most abundant organic acid excreted by all other strains, excretions of citric acid was equivalent or more abundant in LS3 (Additional file 1: Figure S3). In glucose media, in particular, the final concentration of acetic acid was no more than half that of citric acid for this strain. This lower final concentration of acetic acid was due to reaching the plateau early (C/N 90, Additional file 1: Figure S3) or clear reuptake of this compound (C/N 60, Additional file 1: Figure S3), whereas glucose was not exhausted.

In terms of their FA composition, *Blastobotrys* strains synthesized and stored four main FAs during the 96 h of cultivation, accounting for more than 96% of total FA content: C18:1 (43 to 52%, depending on the strain and medium), C16:0 (23 to 27%), C18:2 (11 to 20%), and C18:0 (4 to 11%) (Additional file 1: Figure S4). C16 and C18 FAs are typically the most abundant FAs in a wide range of yeasts and fungi [60, 61]. However, the relative proportion varies depending on the yeast and culture/media conditions. The FA fraction was particularly poor in mono unsaturated C16:1. This feature was also noted for *B. adeninivorans* CBS 8244^T^ in a comparative study of the FA composition of Saccharomycotina yeasts cultivated in YPD [62].

We found consistent but only very low levels of C17 chain-length FA (< 4 mg/g DCW). Indeed, yeast are not known to naturally synthesize large amounts of such odd-chain FAs, unless provided, for example, with odd-carbon precursors or benzoate [63–65]. Olstorpe et al. [17] reported FA profiles for CBS 8244^T^ and CBS 7377 cultured in YPD medium, which were unexpectedly poor in oleic acid (3 to 11% of total FA at 30 °C) and rich in C17:1 (20 to 29% of total FA at 30 °C). The reason for these uncommon profiles is unknown, especially since Froissard et al. [62] reported the same four main FAs in *B. adeninivorans* CBS 8244^T^ cultivated in YPD at 28 °C as those observed here.

### Lipid synthesis from various carbon sources

**Fig. 5:**
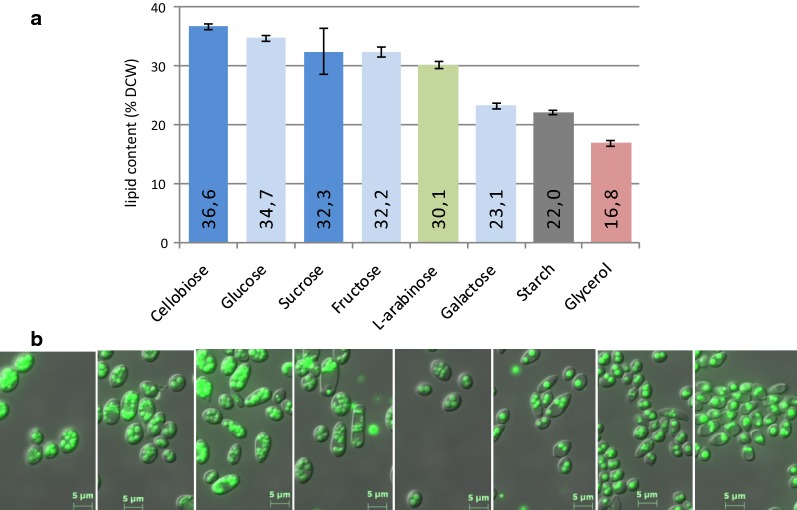
Bioconversion of various sugars into lipids. LS3 was cultivated for 72 h in nitrogen-limited YNB-based medium (0.75 g/L NH_4_Cl) supplemented with the indicated sugars at 30 g/L. **a** Lipid content (% DCW) is presented as a bar with the average value and mean deviation (*n* = 2) and a color code indicating the nature of the carbon source: green, C5 monosaccharide; light blue, C6 monosaccharide; blue, disaccharide; gray, polysaccharide; red, polyol. **b** Microscopic images after BODIPY^®^ staining of the 72 h-cultivated cells in the relevant medium (same order as in the histograms)

Sugars found in plants as reserve sugars or constituting lignocellulosic fibers, as well as the industrial by-product glycerol, are attractive substrates for microbial oil production. We tested the LS3 strain, our best lipid producer, for its ability to accumulate lipids while growing in various hexoses, polysaccharides, or glycerol. The FA fraction of the cells was characterized after 72 and 96 h of culture at 28 °C in YNB-based medium with the C source at 30 g/L and 0.75 g/L NH_4_Cl. After 96 h, lipids accumulated to above 30% DCW in all substrates, except galactose, starch, and glycerol (Fig. 5). Maximum lipid synthesis and storage was obtained for some of the sugars (i.e., cellobiose, glucose, sucrose, or fructose), likely rapidly assimilated, after 96 h in our shake-flask cultures since the rise between 72 and 96 h was less than 5.5% (Additional file 2: Table S1). In contrast, glycerol appeared to be the least favorable substrate for bioconversion into lipids (total FAs reached only 16.1 ± 0.4% DCW), probably because of its slow assimilation, which also affected the OD reached at 96 h: 11.4 ± 1.0% in glycerol instead of 16.8 ± 0.4% in cellobiose. We also observed small differences in the composition of FA profiles relative to that obtained in glucose, especially when LS3 was grown in starch or glycerol (Additional file 2: Table S1). Such variations that depend on the culture conditions have already been reported for other oleaginous yeasts [66]. In all cases, however, C18:1 was the main FA, followed in decreasing proportion by C16:0, C18:2, and C18:0 (Additional file 2: Table S1). In conclusion, all tested substrates, from simple to complex, could be turned into lipids by strain LS3, although with varying efficiency.

### Efficient lipid synthesis at high temperature

*Blastobotrys adeninivorans* and *B. raffinosifermentans*, represented by the strains listed in Table 1, are known to grow at 45 °C. Temperature is known to influence plasma membrane composition, which must adapt to preserve an adequate level of fluidity and function. The nature of FAs, including their chain length and degree of unsaturation (DUS), is involved in this aspect [67–69] and we reasoned that FAs may be stored or recycled accordingly. We thus investigated the ability of two strains, LS3 (*B. raffinosifermentans*) and CBS 8244^T^ (*B. adeninivorans*), to produce lipids at four different temperatures: 28, 37, 42, and 45 °C.

**Fig. 6:**
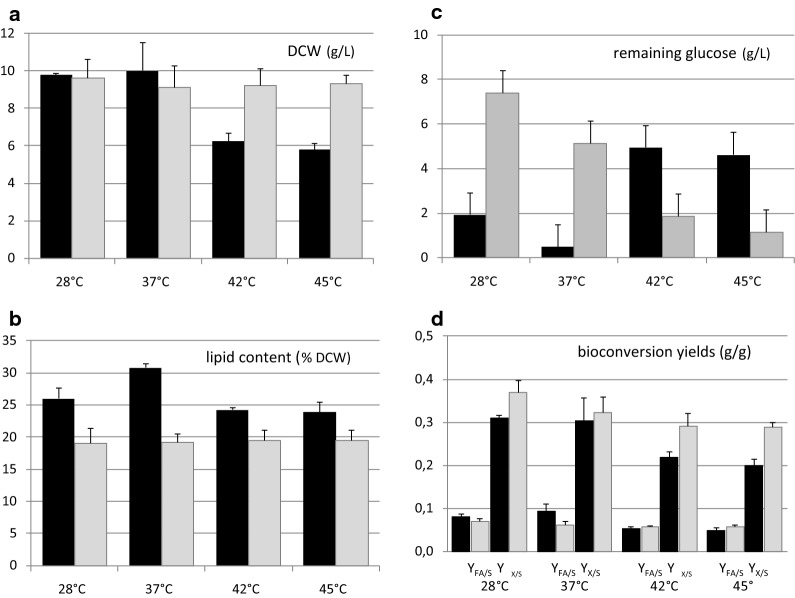
Shake-flask cultures of strains LS3 and CBS 8244^T^ at various temperatures. DCW (**a**), lipid content of the cells (**b**), and glucose concentration in the medium (**c**) were measured after 72 h of cultivation in nitrogen-limited YNB-based medium (30 g/L glucose and 0.75 g/L NH_4_Cl), and the yields (**d**) calculated for strains LS3 (black bars) and CBS 8244^T^ (gray bars). Average values and standard deviations (*n* = 4) are presented in the histograms

Cultures in C/N 60 glucose media were periodically monitored by OD_600_ and microscopy to evaluate the temperature-dependent growth capacity of the strains and possible filamentation. Both strains grew at each temperature tested (Additional file 1: Figure S5). However, growth of LS3 was lower with high temperatures (42 °C and 45 °C), in contrast to that of CBS 8244^T^ (Additional file 1: Figure S5 for OD, Fig. 6a for biomass in g/L). Hyphae were only transiently detected in our lipogenic YNB medium (Additional file 1: Figure S5), contrary to other media, such as YPD, which gradually pushed the LS3 and CBS 8244^T^ cells into (pseudo)hyphae phase above 37 °C and 42 °C, respectively ([11], our unpublished results in YPD medium).

As observed for growth, the amount of total cellular FAs was independent of the temperature of cultivation for CBS 8244^T^: approximately 19% DCW (Fig. 6b). Instead, LS3 showed an optimum at 37 °C for lipid content, reaching 30% DCW (approximately 20% higher than at 28 °C and 25% higher than at 42 to 45 °C). Despite the moderate decrease in lipid content of LS3 at temperatures above 37 °C, we concluded that lipid metabolism of *B. adeninivorans* and *B. raffinosifermentans* was still active at temperatures of up to 45 °C.

Sugar consumption was temperature-dependent for both CBS 8244^T^ and LS3, however, with opposite trends (Fig. 6c). For strain LS3, glucose consumption was lower at elevated temperatures (42 °C and 45 °C), consistent with the lower biomass and lipid content. Instead, glucose consumption of strain CBS 8244^T^ was higher at elevated temperatures, though the amount of lipid and biomass were globally conserved. Overall, final bioconversion yields (*Y*_FA/S_) were within the range of 0.048 to 0.093 for LS3 and 0.056 to 0.070 for CBS 8244^T^ (Fig. 6d and Additional file 2: Table S2). Higher yields were obtained at lower temperatures (28 °C, 37 °C) for both strains; the difference was more pronounced, however, for LS3.

Finally, we examined the FA composition for each strain at each temperature in detail, early in the culture (4.5 h) and at 24 h and 72 h (Additional file 1: Figure S6 and Additional file 2: Table S3). The most divergent FA profiles between the two strains were globally observed at 4.5 h. At that stage, as lipid content of the cells is still low (4 to 8% DCW), structural lipids are likely to significantly contribute to these differences. In this respect, a specific trait of CBS 8244^T^ relative to LS3 is its richness in both C16:0 and C18:2, which is relatively independent of temperature (37 to 45 °C). A comparison of the proportions of FAs at 4.5 and 72 h showed that C18:1 accumulated preferentially over C18:2 in CBS 8244^T^, whereas C16:0 was preferentially stored over C18:0 in LS3. Overall, at 72 h, the DUS of total FAs was slightly lower in CBS 8244^T^ than in LS3, regardless of the temperature (by 6 to 14%).

Temperature had only a moderate effect on global FA composition of the cells. Indeed, we were unable to detect a specific temperature-dependent FA between 28 and 45 °C; the four main FAs remained the same and their relative abundance had only a moderate impact on the global DUS and chain-length index (C16/C18). The maximum temperature-dependent difference of the DUS was 8.5% and that of C16/C18 38% for each strain at each stage of growth. Other authors have also observed that the major FAs in total lipids of various yeast species remained the same over a wide range of temperatures [67, 70, 71], although the DUS and C16/C18 could be altered by more than 25% and 100%, respectively [70, 71]. Their detailed analysis revealed complex alterations in the abundance of FAs and its dependence on temperature, the specific nature of FAs depending on the species (in particular the presence of C18:3 or C16:1) and lipid class (i.e., different categories of phospholipids, triacylglycerols, etc.). In our analysis, which considered only total lipids, the alteration of FA composition, though modest, also appeared to be complex and strain and temperature-dependent. However, elevated temperatures had a similar effect for both CBS 8244^T^ and LS3 for certain FAs at 72 h (Additional file 1: Figure S6). From 28 to 42 °C, C16:0 and C18:1 decreased moderately in proportion to the total lipids, whereas C18:0 and C18:2 increased. Consequently, increasing temperatures led to more similar proportions of C16:0 and C18:2 (likely stored at this stage). The FA profile at 45 °C was highly similar to that at 42 °C for each strain. The strategy of adaptation to ‘high’ temperatures expectedly varies among species and strains; in the aforementioned *R. toruloides* thermotolerant mutant, a temperature increase from 30 to 37 °C, while stimulating lipid production, drastically raised the relative proportion of C18:1, which reached 86% of total FA [32].

In conclusion, lipid metabolism of the LS3 and CBS 8244^T^ strains was robust at temperatures from 28 to 45 °C. Temperature had a moderate impact on the final amount and nature of total cellular FAs, although LS3 showed optimal lipid production at 37 °C. Such robustness of lipid metabolism to differences in temperature is not shared across all yeasts. For example, the lipid content of *L. starkeyi* DSM 70295 reportedly decreased by 57% when the culture temperature was raised from 10 to 20 °C [70]. In the thermophilic species *Kluyveromyces marxianus*, 11 wild-type strains recently screened for lipogenesis (the best strain reportedly reached 10% DCW, mainly stored as free FAs) had a much lower FA content at 30 than 42 °C [42].

## Conclusions

Harnessing microbial lipid metabolism to produce oils or other chemicals of interest is a promising alternative to petroleum or plant oil. However, limitations in the use of yeast have been encountered, such as the poor utilization of certain types of substrates, weak xerotolerance, limited yields for lipid conversion in competition with other metabolic pathways, or difficult culturing in bioreactors (filamentation, foaming). We believe that the availability of a set of various oleaginous microorganisms adapted to different types of applications could be useful. Elevated temperatures may help reduce the costs of the process and participate in the solubilization of substrates. *B. adeninivorans* and *B. raffinosifermentans* exhibit many advantageous traits for biotechnological processes and, as reported here, were able to synthesize and store lipids at high temperatures, up to 45 °C.

Among four tested strains, LS3 was the best lipid-producing one. Although it showed a temperature dependence for growth, glucose consumption, and lipid storage, its native performance in the tested range of 28 to 45 °C is a good starting point (24 to 30% DCW from glucose).

As for other oleaginous yeasts, strain screening, process optimization, and/or metabolic engineering is likely to be successful. Knowledge of the lipid metabolism in these *Blastobotrys* species, as well as their genetic engineering, could be facilitated by their small genome. Accurate genome editing tools should be developed to complement existing classical genetic tools.

## Materials and methods

### Strains, genotyping, identification

Yeast strains formerly classified as *B. adeninivorans* are listed in Table 1; LS3 was in the collection of IPK, other strains were obtained from the CBS-KNAW. *Y. lipolytica* W29 was used as a control [72].

Genotyping of these strains was performed by intergenic spacer rDNA amplification and *Alu*I Fingerprinting (IGSAF). The IGSAF technique has been successfully used to differentiate previously misidentified species in different taxa [73, 74].

The entire ITS + D1D2 region of the rDNA was amplified from genomic DNA in a single run, as previously described [75], using the primer pair ITSF (5′ AGGAACTAAAAGTCGTAACAAG) and DDR (5′ GGTTTTACACCCAAACACTC). After sequencing, the ITS and D1D2 sequences were delimited before multiple alignment with clustal X [76]. Sequences used for strain identification were as follows: ITS-D1D2 for LS3 was extracted from the LS3 genome sequence  in the GRYC database [77] and mitochondrial *COXII* was retrieved from the NCBI accession number CBZY010000007; ITS-D1D2 fragments for CBS 8244^T^, CBS 7350, CBS 7766, CBS 8335, CBS 7377, CBS 7370, and mitochondrial mtCOXII (partial sequence) for CBS 7370 were deposited at the ENA under the study accession number PRJEB29673; type strain sequences for the mtCOXII marker were retrieved via YeastIP and the Genbank accession numbers were DQ443105.1 (CBS 6800^T^) and DQ443104.1 (CBS 8244).

### BLAST search

Homologous gene products shared by *Y. lipolytica* E150 and *B. raffinosifermentans* LS3 were sought by reciprocal BLASTP with the BLOSUM62 matrix, using facilities of the GRYC database [77] and a threshold of 1.e^−40^.

### Media and growth conditions

Rich medium YPD (yeast extract, peptone, and glucose, 10 g/L each) was used for precultures and minimal medium (YNB) supplemented with various carbon sources for cultures. All YNB-based media contained 1.7 g/L yeast nitrogen base (without amino acids or ammonium sulfate, Difco, Paris), NH_4_Cl as the nitrogen source (at various concentrations, as indicated), and were buffered with 50 mM phosphate buffer (Na_2_HPO_4_, KH_2_PO_4_, pH 6.8). Cells were cultured at 28 °C unless otherwise indicated. Absorbance at 600 nm (OD_600_) was measured with an LKB-Novaspec II spectrophotometer (Pharmacia), except for growth tests in microplates.

Growth tests were carried out in 96-well microplates (Greiner) incubated in a BIOTEK synergy MX plate reader under strong and constant agitation. OD_600_ was measured every 20 min. Wells were filled with 190 µL YNB-based buffered media with glucose, xylose, or glycerol (at either 10 or 2 g/L), all supplemented with 5 g/L NH_4_CL. They were inoculated with 10 µL of a cell suspension from overnight precultures in YPD and adjusted to an OD_600_ of 4 by the addition of the desired volume of YNB (without carbon) to the cell pellet.

### Cultures for lipid accumulation

Experiments were carried out in 500-mL baffled flasks filled with 100 mL YNB-based medium and incubated under agitation at 160 rpm in a Minitron incubator (INFOR-HT, Swisserland). Carbon sources were added at a concentration of 30 g/L. When indicated, the carbon-to-nitrogen mass ratio (C/N) was adjusted to 60 or 90 by adding 0.75 g/L or 0.5 g/L NH_4_Cl, respectively. Oleic acid (65.0–88.0%, Sigma-Aldrich) used in this study contained 70% C18:1 (*n* − 9) *cis*, 3% C18:1 (*n* − 9) *trans*, 7% C18:2 (*n* − 6), 1.2% C17:1, 3.9% C16:0, 4.7% C16:1 (*n* − 7), 0.9% C16:1 (*n* − 9), and other FAs in low amounts [78]. Cultures were routinely performed at 28 °C. The experiment testing various temperatures was carried out for 72 h in four incubators in which the temperature was recorded by USB thermometers.

Precultures were grown for 20 h at 28 °C in flasks filled with YPD and used for culture inoculation at an OD_600nm_ of 0.5. YPD of the preculture was eliminated by centrifugation and the cells resuspended in YNB without carbon and nitrogen.

### Lipid and biomass quantification

Lipid content of the cells was determined by quantifying their FA fraction, as previously described [59]. Cells from-10 mL culture samples were collected in pre-weighed tubes, washed twice in water (for cultures in sugar-based medium), or twice successively in a mixture of 0.5% BSA and 0.9% NaCl and once in water (for oily medium), and resuspended in 1 mL water. After freeze-drying for 24 h at − 55 °C (Alpha 1-2Dplus, Bioblock Scientific), samples in their tubes were weighed and stored at − 20 °C. The difference in tube weights represented the mass of the cells found in 10-mL cultures and permitted calculation of the biomass in g/L.

Fatty acid methyl esters (FAMES) were recovered from 10 to 20 mg aliquots of freeze-dried cells (2 to 4 mg in samples collected at 4.5 h), using a hot methanol–H_2_SO_4_ method, adapted from Browse et al. [79], and analyzed by gas chromatography on a Varian 430 equipped with a flame-ionization detector and a FactorFour vf-23 ms column. FAMES were identified by comparison to commercial standards (FAME32; Supelco); an internal FA standard (100 µg C12:0 from Sigma) was added to each sample prior to transesterification to enable FA quantification in the analyzed aliquots.

Lipid content is expressed with respect to DCW: lipid content of 1% DCW = 10 mg FA per g of dry cell.

### FA profiles, index, statistics

The main identified FAs were: C18:1 (*n* − 9) *cis* (oleic acid, simplified as C18:1 in the text), C18:0 (stearic acid), C18:2 (*n* − 6) *cis* (linoleic acid, simplified as C18:2 in the text), and C16:0 (palmitic acid). C16:1 (*n* − 9) *cis* (palmitoleic acid) and C16:1 (*n* − 7), both grouped under C16:1, were also identified, in low amounts. Unidentified or minor FAs were grouped under ‘other FAs’; among them, C18:3 was sporadically detected (relative amount < 0.45%).

The DUS [69] was calculated as follows: (percentage of monoene FAs + 2 × the percentage of diene FAs)/total percentage of four main FAs. C16/C18 [70] was calculated as the ratio of carbon-chain length of 16 to carbon-chain length of 18 for the four main FAs.

Kruskal and Wallis tests with multiple comparisons [80] were run using Excel facilities designed by P. Georgin, M. Gouet, and G. Le Pape.

### Quantification of sugars and organic acids

Sugars and organic acids in the culture supernatants were analyzed by the HPLC method of calibration with external standards. Immediately after centrifugation of culture samples, supernatants were conserved frozen (− 20 °C). Prior to analysis, they were homogenized, centrifuged again to eliminate any remaining cells, and diluted 10 times in water. They were analyzed by HPLC (UltiMate 3000, Dionex-ThermoFisher Scientific, UK) coupled to UV (210 nm) and RI detectors. The Aminex HPX87H column (Thermo Scientific, Waltham, MA) was eluted with 0.01 N H_2_SO_4_ at 35 °C at a flow rate of 0.6 mL/min. Peak integration, identification, and quantification were performed using CHROMELEON software (Thermo Scientific, USA) and standards purchased at Sigma-Aldrich.

### Cell imaging

Images were acquired using a Zeiss Axio Imager M2 microscope (Zeiss, Le Pecq, France) fitted with an Apochromat 100×/1.40 oil M27 objective and operated with Axiovision 4.8.2 software. Fluorescently-stained lipid bodies were visualized using Zeiss filters 45 and 46, after incubating culture samples with 1.5 µg/mL BODIPY^®^ Lipid Probe (493/503 (D-3922), Invitrogen) for at least 15 min at room temperature.

## Additional files

**Additional file 1: Figure S1.** Polymorphisms in the ITS-D1D2 nucleotide sequence. **Figure S2.** Growth of *B. adeninivorans* and *B. raffinosifermentans* in three different substrates. **Figure S3.** Organic acids excreted in lipogenic culture conditions. **Figure S4.** FA composition of strains of *B. raffinosifermentans* and *B. adeninivorans* after growth in lipogenic medium. **Figure S5.** Growth curves and microscopic images of strains LS3 and CBS 8244^T^ cultured in glucose lipogenic medium at various temperatures. **Figure S6.** Relative FA composition of strains LS3 and CBS 8244^T^ at various times and temperatures of cultivation in glucose lipogenic medium.

**Additional file 2: Table S1.** Relative composition (%) of the FA fraction of LS3 cells after growth in various substrates. **Table S2.** Final yields after 72 h of cultivation of LS3 and CBS8244^T^ in YNB-based glucose media (C/N = 60) at different temperatures. **Table S3.** Time course analysis of the FA profiles of strains LS3 and CBS 8244^T^ cultivated in YNB-based glucose media (C/N = 60) at various temperatures.

## Data Availability

All data generated or analyzed during this study are included in this published article and its additional files.
