# Supplementary material for: Blastobotrys adeninivorans and B. raffinosifermentans, two sibling yeast species which accumulate lipids at elevated temperatures and from diverse sugars
Source: Biotechnol Biofuels. 2019 Jun 20;12:154. doi: 10.1186/s13068-019-1492-x (PMC6587252; doi:10.1186/s13068-019-1492-x)
Supplement: Supplementary file 1 — Additional file 1: Figure S1. Polymorphisms in the ITS-D1D2 nucleotide sequence. Figure S2. Growth of B. adeninivorans and B. raffinosifermentans in three different substrates. Figure S3. Organic acids excreted in lipogenic culture conditions. Figure S4. FA composition of strains of B. raffinosifermentans and B. adeninivorans after growth in lipogenic medium. Figure S5. Growth curves and microscopic images of strains LS3 and CBS 8244T cultured in glucose lipogenic medium at various temperatures. Figure S6. Relative FA composition of strains LS3 and CBS 8244T at various times and temperatures of cultivation in glucose lipogenic medium. [file 13068_2019_1492_MOESM1_ESM.pdf]

```
                               1 1
                             230 0
                           1 620 4
                           8 719 0
CBS 8244  TTGTCTTAGGACATTTTT/GTG/AGCTGGGACCGAGGACTGCGCATCTGCTAGGATGTTGGCGTAATGAC
CBS 7350         A         T GTG A
CBS 7766         C         T GCG A
LS3              C         - ACA T
CBS 7370         C         - ACA T
CBS 7377         C         - ACA T
CBS 8335         C         - ACA T
```

**Figure S1. Polymorphisms in the ITS-D1D2 nucleotide sequence.**

For each of the seven strains, a fragment spanning rDNA ITS-D1D2 was PCR-amplified. Their sequences were multi-aligned and restricted to a common fragment of non ambiguous 1086 nt. The sequence of the reference strain (CBS8244$^{T}$) is indicated as follow:  it is in extent from nt 1 to 8 and from nt 1040 to 1086; within slashes, only sites of polymorphism are shown with their coordinates at the top, reading downward. SNP are indicated below for other strains, the hyphen representing a single nt deletion.

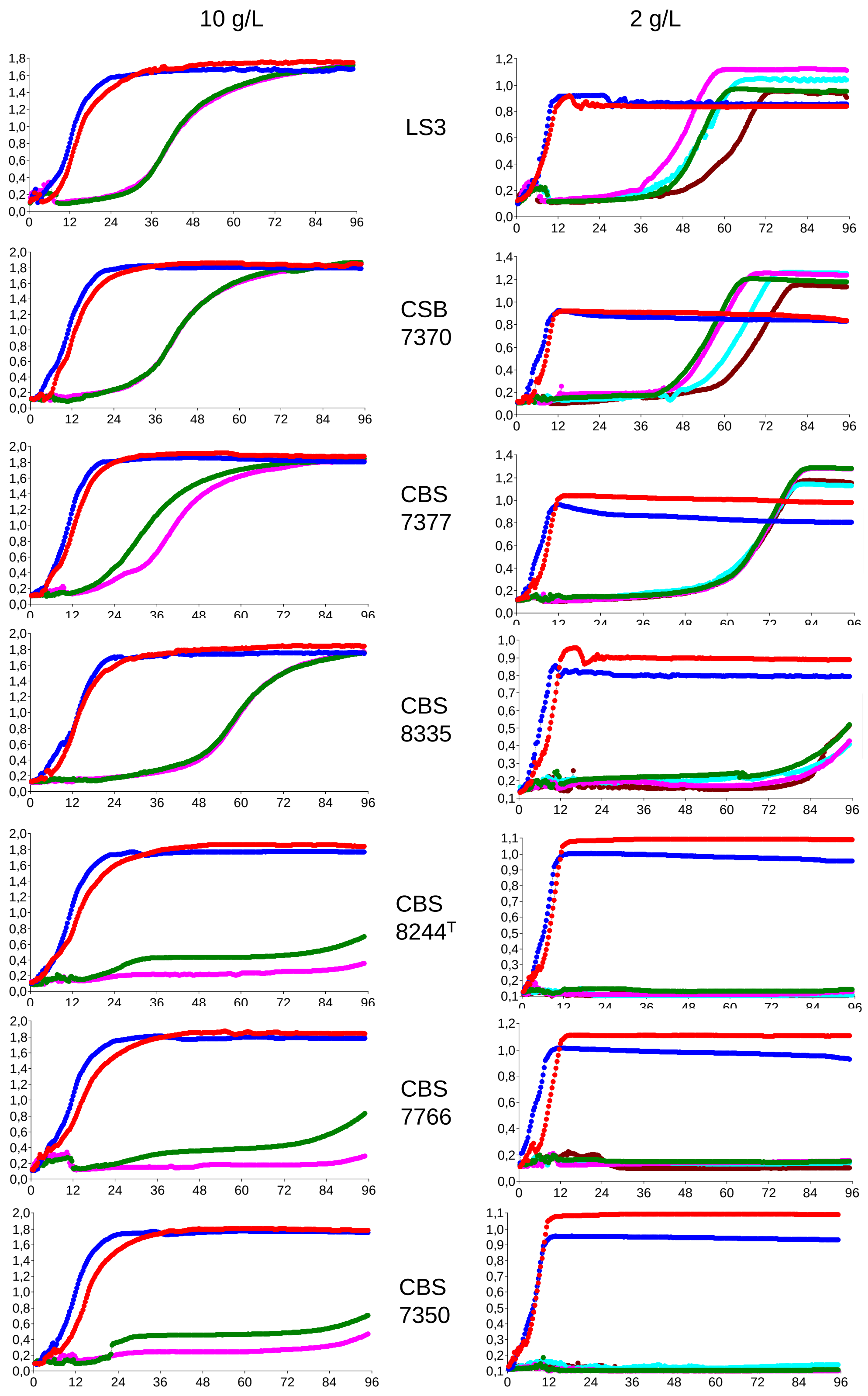


**Figure S2. Growth of *B. adeninivorans* and *B. raffinosifermentans* in three different substrates.**
Representative growth curves plotting $OD_{600}$ against time (h) are shown; several curves were selected for growth in glycerol to give insights into the observed variability. Xylose (red curves), glucose (dark blue curves) and glycerol (other colors) were used at 10 g/L (left panel) or 2 g/L (right panel) to cultivate the 7 strains in 96-well microplates. Upper four strains constitute the group reassigned to *B. raffinosifermentans*. Note that max OD cannot be correctly resolved at a concentration of substrate of 10 g/L due to intrisinc limitation of micoplate reader.

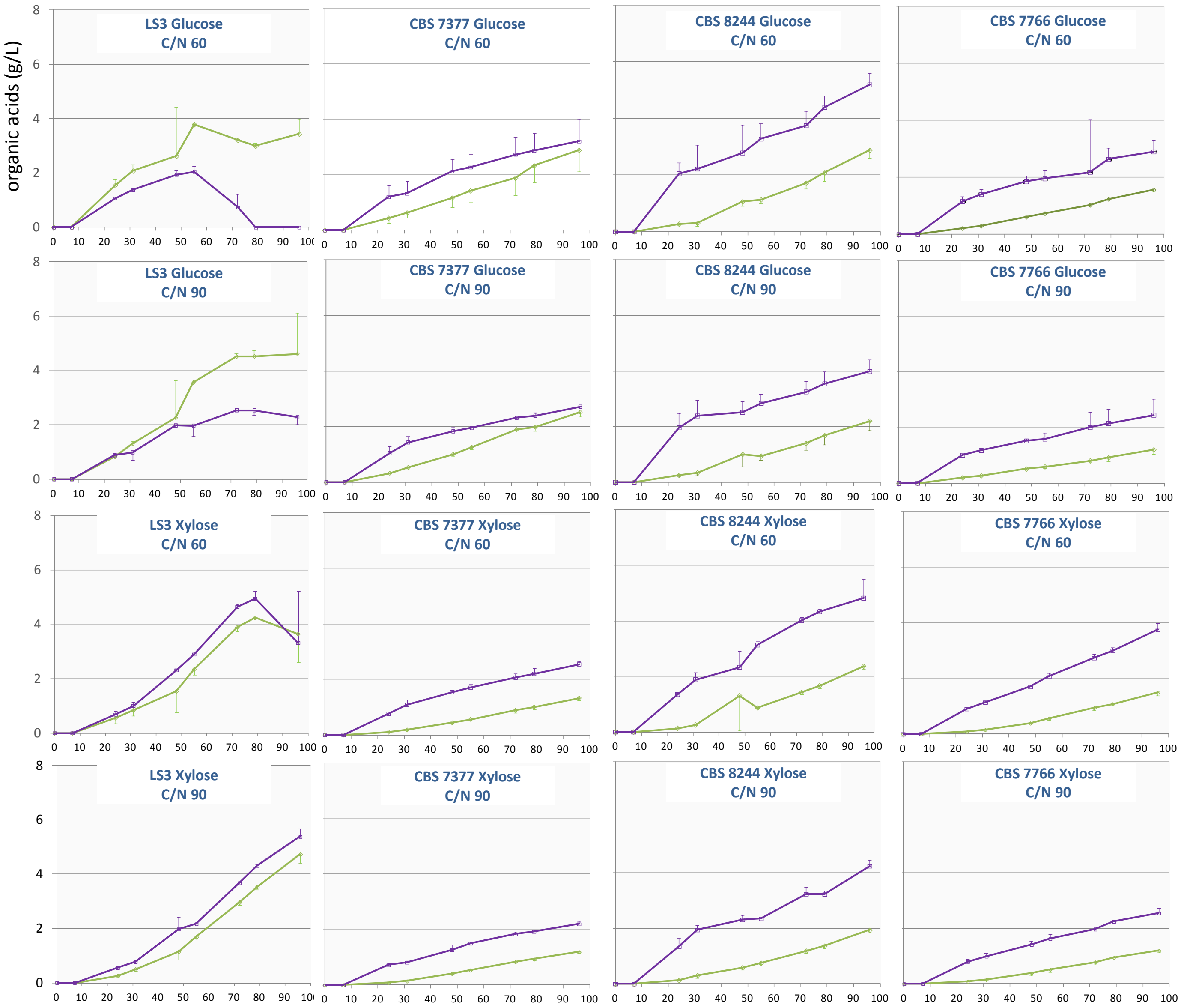


**Figure S3. Organic acids excreted in lipogenic culture conditions.**
The indicated strains were grown in 30 g/L glucose or xylose at two C/N ratios. The concentration of acetic acid (purple line) and citric acid (green line) in culture supernatants (average values and standard deviations, n=3) is plotted over time (h).

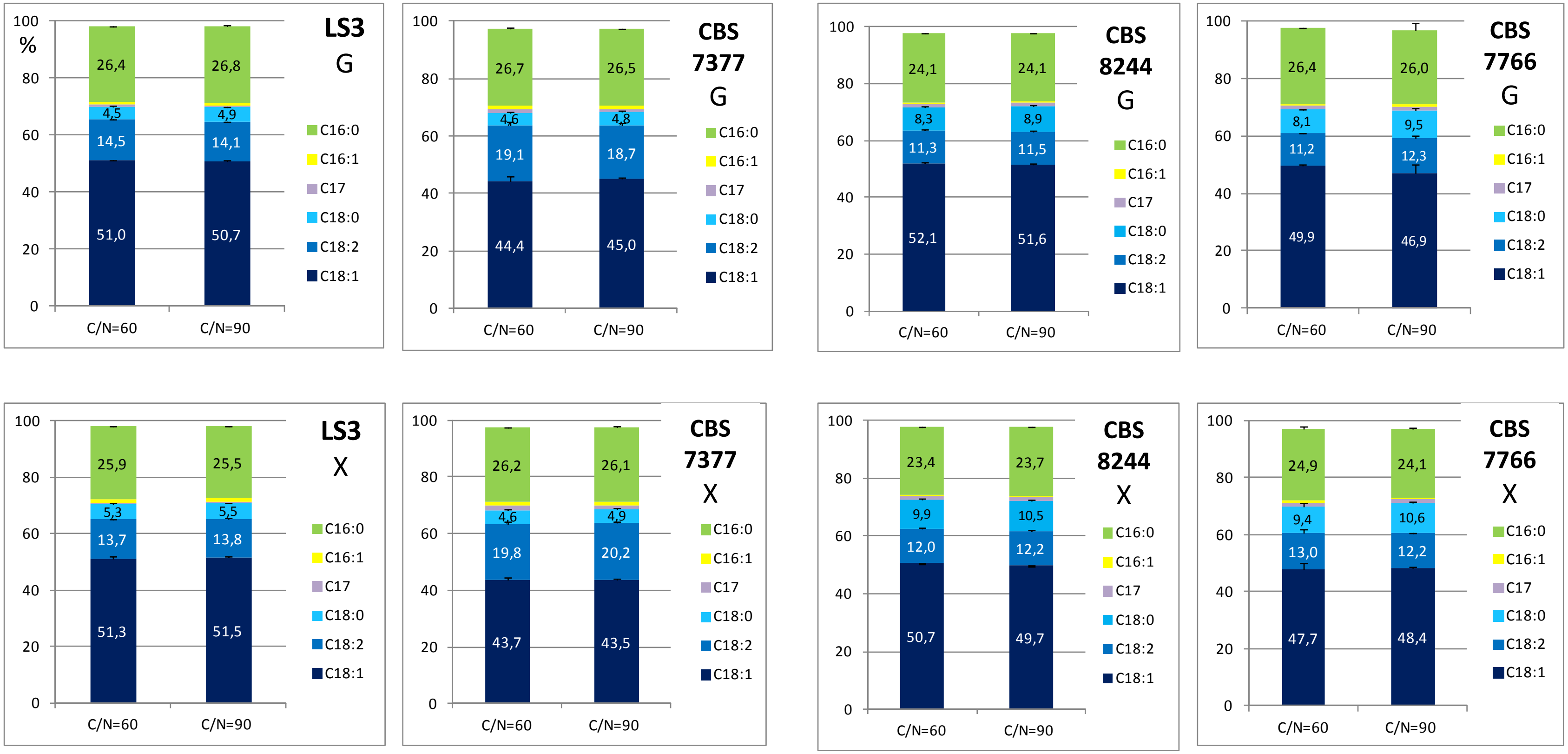


**Figure S4. FA composition of strains of *B. raffinosifermentans* and *B. adeninivorans* after growth in lipogenic medium.**
The indicated strains were grown for 96h in 30 g/L glucose (G) or xylose (X) at two C/N ratios. The proportion of the different FAs is given in percentage of the total FA fraction.

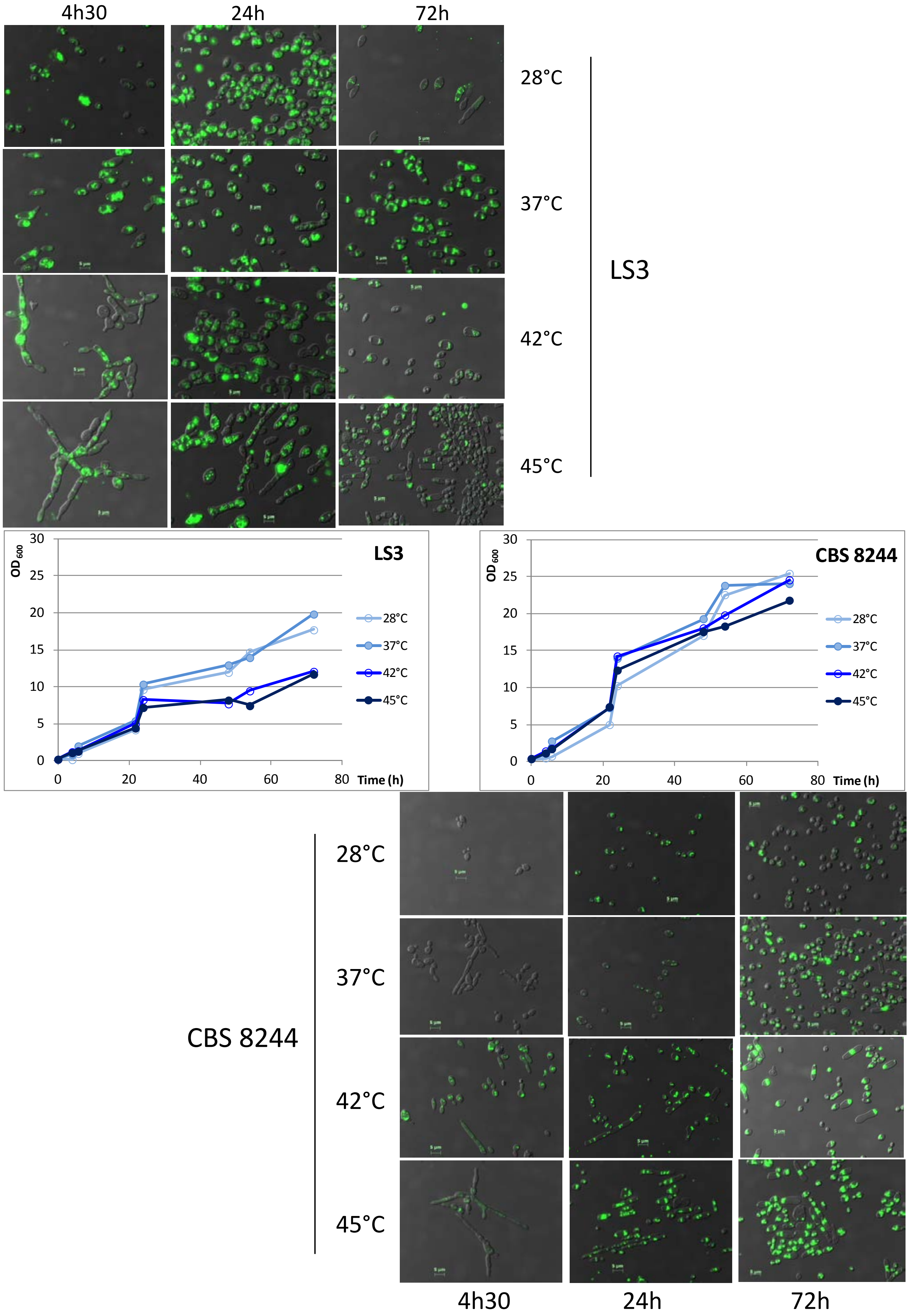


**Figure S5. Growth curves and microscope images of strains LS3 and CBS 8244$^T$ cultured in glucose lipogenic medium at various temperatures.** Neutral lipids were colored with BODIPY.

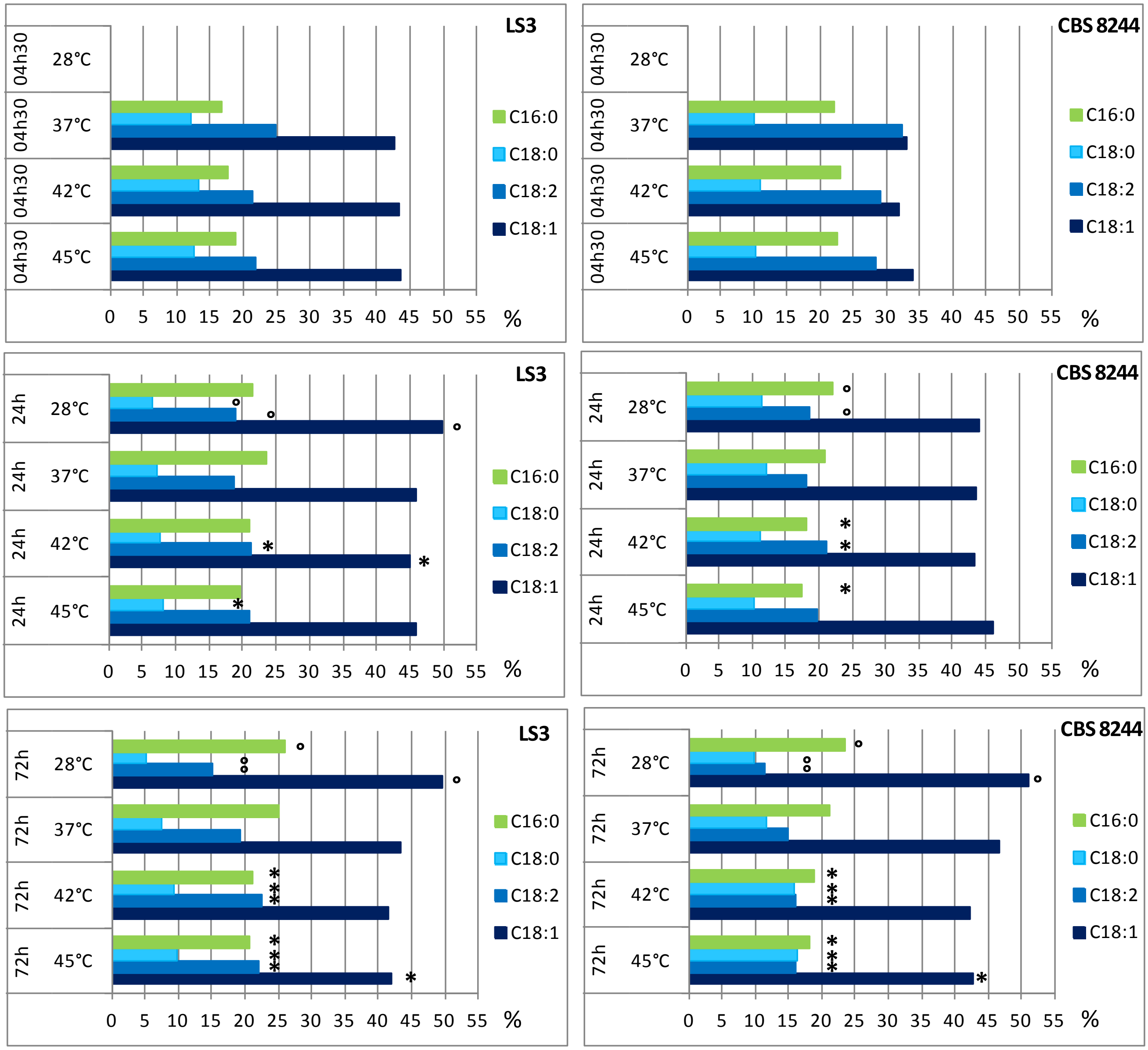


**Figure S6. Relative FA composition of strains LS3 and CBS 8244$^T$ at various times and temperatures of cultivation in glucose lipogenic medium.**

Percentage of total FA fraction (average values, n ≥ 3) is represented as a color bar for each of the four main FAs. See Table S3 for complete data. (*) Indicates significative difference for each FA relative to the reference sample at 28°C (°) in a Kruskal&Wallis test (P<0.05).
