# Supplementary material for: Blastobotrys adeninivorans and B. raffinosifermentans, two sibling yeast species which accumulate lipids at elevated temperatures and from diverse sugars
Source: Biotechnol Biofuels. 2019 Jun 20;12:154. doi: 10.1186/s13068-019-1492-x (PMC6587252; doi:10.1186/s13068-019-1492-x)
Supplement: Supplementary file 2 — Additional file 2: Table S1. Relative composition (%) of the FA fraction of LS3 cells after growth in various substrates. Table S2. Final yields after 72 h of cultivation of LS3 and CBS8244T in YNB-based glucose media (C/N = 60) at different temperatures. Table S3. Time course analysis of the FA profiles of strains LS3 and CBS 8244T cultivated in YNB-based glucose media (C/N = 60) at various temperatures. [file 13068_2019_1492_MOESM2_ESM.pdf]

Table S1. Relative composition (%) of the FA fraction of LS3 cells after growth in various substrates.

Substrate	Time	Lipid content (% DCW)	C18:1	C16:0	C18:2	C18:0	Other FAs
Cellobiose	72h	34.8 ± 1.2	45.1 ± 0.6	28.1 ± 0.2	16.8 ± 0.4	6.1 ± 0.1	3.8 ± 0.1
	96h	36.6 ± 0.5	45.5 ± 0.8	27.8 ± 0.0	17.1 ± 0.3	6.0 ± 0.1	3.5 ± 0.5
Glucose	72h	32.9 ± 1.2	48.8 ± 0.4	27.4 ± 0.3	14.7 ± 0.22	5.4 ± 0.3	3.7 ± 0.2
	96h	34.7 ± 0.5	48.5 ± 0.3	27.4 ± 0.3	15.0 ± 0.1	5.3 ± 0.3	3.9 ± 0.2
Sucrose	72h	30.7 ± 2.2	49.6 ± 1.5	26.4 ± 0.1	14.7 ± 1.23	5.4 ± 0.1	3.9 ± 0.3
	96h	32.3 ± 3.9	49.2 ± 1.2	26.5 ± 0.1	14.9 ± 1.1	5.1 ± 0.1	4.3 ± 0.2
Fructose	72h	31.9 ± 0.2	51.3 ± 0.5	26.1 ± 0.1	13.8 ± 0.5	5.3 ± 0.1	3.4 ± 0.1
	96h	32.2 ± 0.9	50.0 ± 1.0	25.9 ± 0.1	14.1 ± 0.3	5.3 ± 0.1	4.6 ± 0.7
L-arabinose	72h	27.3 ± 1.1	44.0 ± 0.1	26.7 ± 0.4	19.7 ± 0.0	4.5 ± 0.1	5.1 ± 0.6
	96h	30.1 ± 0.6	48.0 ± 3.2	26.8 ± 0.9	16.1 ± 2.3	4.9 ± 0.4	4.3 ± 0.4
Galactose	72h	18.6 ± 1.6	50.7 ± 0.8	25.2 ± 0.1	15.3 ± 0.2	4.6 ± 0.0	4.1 ± 0.6
	96h	23.1 ± 0.6	50.9 ± 0.5	25.1 ± 0.2	14.7 ± 0.2	4.7 ± 0.1	4.5 ± 0.0
Starch	72h	19.3 ± 2.1	55.1 ± 0.9	21.9 ± 0.5	12.1 ± 0.6	6.7 ± 0.4	4.1 ± 0.4
	96h	22.0 ± 0.4	55.1 ± 0.1	21.9 ± 0.1	12.1 ± 0.2	5.9 ± 0.3	4.9 ± 0.1
Glycerol	72h	13.2 ± 0.3	46.1 ± 0.1	19.2 ± 0.0	22.3 ± 0.4	6.3 ± 0.1	6.0 ± 0.4
	96h	16.8 ± 0.4	47.3 ± 1.2	19.4 ± 0.4	20.4 ± 0. 7	6.0 ± 0.1	7.0 ± 2.3

Average values and mean deviations (n=2) are indicated.

Cells were cultivated in N-limited YNB-based media for the indicated time.

Table S2. Final yields after 72 h of cultivation of LS3 and CBS 8244[T] in YNB-based glucose media (C/N = 60) at different temperatures.

		Lipid titer g/L	DCW g/L	$Y_{FA/S}$	$Y_{X/S}$
**LS3**	**28°C**	2.54 ± 0.16	9.78 ± 0.10	0.080 ± 0.007	0.310 ± 0.006
	**37°C**	3.08 ± 0.49	10.00 ± 1.49	0.093 ± 0.017	0.303 ± 0.053
	**42°C**	1.51 ± 0.12	6.22 ± 0.43	0.053 ± 0.004	0.218 ± 0.014
	**45°C**	1.38 ± 0.17	5.77 ± 0.34	0.048 ± 0.006	0.200 ± 0.013
**CBS 8244[T]**	**28°C**	1.83 ± 0.28	9.62 ± 1.00	0.070 ± 0.006	0.369 ± 0.027
	**37°C**	1.76 ± 0.28	9.12 ± 1.13	0.062 ± 0.007	0.322 ± 0.035
	**42°C**	1.78 ± 0.11	9.20 ± 0.91	0.056 ± 0.003	0.291 ± 0.029
	**45°C**	1.82 ± 0.17	9.32 ± 0.46	0.056 ± 0.004	0.289 ± 0.011

Average values and standard deviations (n = 4)

Table S3. Time course analysis of the FA profiles of strains LS3 and CBS 8244^T cultivated in YNB-based glucose media (C/N = 60) at various temperatures.

LS3

		Lipid content (% DCW)	C18:1	C16:0	C18:2	C18:0	C17:0	Other FAs	DUS	C16/C18	
**4h30**	**28°C**	6.2 ± 1.5	35.3 ± 0.9	19.0 ± 0.5	36.5 ± 1.5	9.1 ± 0.1	0.0	0.0	nd	nd	(n=2)*
	**37°C**	7.3 ± 1.4	42.7 ± 0.7	16.8 ± 0.3	24.9 ± 4.2	12.2 ± 1.9	1.0 ± 0.6	2.7 ± 1.3	0.95	0.21	(n=3)
	**42°C**	8.1 ± 1.8	43.4 ± 2.9	17.6 ± 0.6	21.3 ± 1.3	13.3 ± 1.3	0.9 ± 0.2	3.4 ± 2.9	0.89	0.23	
	**45°C**	7.4 ± 1.5	43.7 ± 2.7	18.7 ± 1.0	21.8 ± 0.8	12.7 ± 0.5	1.0 ± 0.3	2.2 ± 1.5	0.89	0.24	(n=3)
**24h**	**28°C**	14.0 ± 1.7	49.8 ± 1.3	21.4 ± 1.0	19.0 ± 0.9	6.5 ± 0.3	0.5 ± 0.1	2.8 ± 0.4	0.90	0.28	
	**37°C**	18.0 ± 1.4	45.9 ± 0.3	23.6 ± 0.9	18.8 ± 0.7	7.2 ± 0.5	0.8 ± 0.1	3.7 ± 0.3	0.87	0.33	
	**42°C**	16.3 ± 1.6	45.0 ± 0.8	21.1 ± 0.4	21.2 ± 0.6	7.7 ± 0.2	1.0 ± 0.1	4.1 ± 0.9	0.91	0.29	
	**45°C**	13.0 ± 0.9	46.0 ± 1.2	19.8 ± 0.9	21.1 ± 0.4	8.0 ± 0.1	1.0 ± 0.1	4.1 ± 0.2	0.92	0.26	
**72h**	**28°C**	26.0 ± 1.7	49.6 ± 0.9	26.0 ± 0.6	15.1 ± 0.3	5.2 ± 0.3	0.6 ± 0.1	3.5 ± 0.1	0.83	0.37	
	**37°C**	30.8 ± 0.7	43.3 ± 0.6	25.1 ± 0.5	19.2 ± 0.5	7.5 ± 0.2	0.9 ± 0.1	3.9 ± 0.4	0.85	0.36	
	**42°C**	24.3 ± 0.3	41.5 ± 0.5	21.0 ± 0.2	22.4 ± 0.4	9.3 ± 0.3	1.3 ± 0.2	4.4 ± 0.6	0.90	0.29	
	**45°C**	23.9 ± 1.6	42.1 ± 0.3	20.7 ± 0.2	22.0 ± 0.2	9.7 ± 0.1	1.2 ± 0.1	4.2 ± 0.3	0.90	0.38	

CBS 8244^T

		Lipid content (% DCW)	C18:1	C16:0	C18:2	C18:0	C17:0	Other FAs	DUS	C16/C18	
**4h30**	**28°C**	7.1 ± 3.4	27.4 ± 2.1	28.8 ± 8.9	30.1 ± 17.2	11.7 ± 3.5	0.0	2.0 ± 2.8	nd	nd	(n=2)*
	**37°C**	4.4 ± 0.8	33.1 ± 2.9	22.2 ± 0.8	32.5 ± 3.1	10.1 ± 0.8	1.0 ± 0.2	1.7 ± 1.6	1.00	0.29	
	**42°C**	4.9 ± 1.8	31.9 ± 2.6	23.0 ± 1.2	29.2 ± 6.3	10.9 ± 4.6	1.2 ± 0.1	4.1 ± 3.9	0.94	0.32	
	**45°C**	5.1 ± 1.1	34.0 ± 2.0	22.6 ± 0.4	28.6 ± 2.9	10.2 ± 1.5	1.8 ± 0.7	2.9 ± 2.1	0.94	0.31	
**24h**	**28°C**	8.0 ± 1.1	44.0 ± 0.4	22.0 ± 0.5	18.5 ± 0.6	11.4 ± 0.3	1.1 ± 0.4	2.9 ± 0.6	0.84	0.30	
	**37°C**	10.4 ± 1.5	43.6 ± 0.7	21.0 ± 0.8	18.0 ± 0.4	12.0 ± 0.3	1.4 ± 0.3	3.9 ± 0.6	0.83	0.29	
	**42°C**	9.2 ± 0.3	43.3 ± 0.3	18.2 ± 0.8	21.1 ± 0.8	11.2 ± 1.0	1.4 ± 0.2	4.7 ± 0.8	0.90	0.24	
	**45°C**	8.9 ± 0.4	46.1 ± 1.5	17.4 ± 0.5	19.7 ± 0.5	10.3 ± 1.5	1.2 ± 0.1	5.4 ± 0.2	0.90	0.23	
**72h**	**28°C**	19.0 ± 2.4	51.0 ± 0.7	23.6 ± 0.4	11.5 ± 0.5	9.8 ± 0.4	1.0 ± 0.1	3.2 ± 0.2	0.76	0.33	
	**37°C**	19.2 ± 1.3	46.6 ± 0.3	21.1 ± 0.4	14.8 ± 0.5	11.8 ± 0.2	1.5 ± 0.1	4.1± 0.2	0.79	0.29	
	**42°C**	19.5 ± 1.6	42.3 ± 0.8	18.9 ± 0.4	16.2 ± 0.4	15.8 ± 0.6	1.7 ± 0.1	5.1 ± 0.4	0.80	0.25	
	**45°C**	19.5 ± 1.5	42.7 ± 0.6	18.1 ± 0.4	16.0 ± 0.4	16.3 ± 0.6	1.6 ± 0.1	5.3 ± 0.3	0.76	0.24	

* Tables give average values and standard deviations for n = 4 unless otherwise indicated. The relative proportion (%) of FAs in the profiles as well as index for degree of unsaturation and chain length are indicated. nd, not determined.
